# Apocynin and Hyperbaric Oxygen Therapy Improve Renal Function and Structure in an Animal Model of CKD

**DOI:** 10.3390/biomedicines12122788

**Published:** 2024-12-09

**Authors:** Andrija Vukovic, Danijela Karanovic, Nevena D Mihailovic-Stanojevic, Zoran Miloradovic, Predrag Brkic, Maja Zivotic, Jelena Nesovic Ostojic, Milan Ivanov, Sanjin Kovacevic, Una-Jovana Vajic, Djurdjica Jovovic, Silvio R. De Luka

**Affiliations:** 1 Institute of Pathological Physiology, Faculty of Medicine, University of Belgrade, Dr Subotića 1, 11000 Belgrade, Serbia; andrija.vukovic@med.bg.ac.rs (A.V.); jelena.nesovic-ostojic@med.bg.ac.rs (J.N.O.); sanjin.kovacevic@med.bg.ac.rs (S.K.); 2 Institute for Medical Research, National Institute of Republic of Serbia, University of Belgrade, Dr Subotića 4, 11000 Belgrade, Serbia; danijela.karanovic@imi.bg.ac.rs (D.K.); nevena@imi.bg.ac.rs (N.D.M.-S.); zokim@imi.bg.ac.rs (Z.M.); ivmilan@imi.bg.ac.rs (M.I.); unajovana@imi.bg.ac.rs (U.-J.V.); djurdjica@imi.bg.ac.rs (D.J.); 3 Institute of Medical Physiology, Faculty of Medicine, University of Belgrade, Višegradska 26, 11000 Belgrade, Serbia; predrag.brkic@med.bg.ac.rs; 4 Institute of Pathology, Faculty of Medicine, University of Belgrade, Dr Subotića 1, 11000 Belgrade, Serbia; maja.zivotic@med.bg.ac.rs

**Keywords:** CKD, apocynin, hyperbaric oxygen therapy, 5/6 nephrectomy, fibrosis, desmin, fibronectin, hypoxia-inducible factor-1α

## Abstract

Background/Objectives: Chronic kidney disease (CKD) is a progressive pathological condition which results in the severe fibrosis of the kidneys. However, the mechanisms of CKD progression and fibrogenesis remain unclear. We wanted to examine the effects that apocynin and hyperbaric oxygen therapy (HBOT) have on renal function and structure in animals with CKD induced through 5/6 nephrectomy (5/6 Nx-L). Methods: Male Wistar rats were divided in 5 groups (n = 8/group) as follows: control—sham-operated rats; Nx-L—rats with 5/6 Nx-L; APO—5/6 Nx-L + apocynin treatment; HBOT—5/6 Nx-L + hyperbaric oxygen treatment, and APO+HBOT—5/6 Nx-L, treated with both treatments. All treatments started 4 weeks after the final step of CKD induction and lasted for 4 weeks. At the end of the experiment, urine samples were collected for the proteinuria assessment and the mean arterial pressure (MAP) was measured. Kidneys were collected for histopathological, Western blot, and immunohistochemical analyses. Results: All treatments significantly decreased MAP compared to the Nx-L group (*p* < 0.001). In the APO and APO+HBOT groups, the level of proteinuria was decreased compared to the Nx-L group (*p* < 0.05 and *p* < 0.01, respectively). All examined treatments significantly decreased the intensity of lesions in the kidney compared to those observed in the Nx-L group (*p* < 0.001). Isolated treatments with apocynin and HBOT induced a significant decrease in desmin expression compared to the Nx-L group (*p* < 0.05); meanwhile, they did not affect the levels of fibronectin (FN) and hypoxia-inducible factor-1α (HIF-1α). Combined treatment did not affect desmin expression levels; however, it induced a significant increase in fibronectin expression compared to Nx-L (*p* < 0.001). Conclusions: Apocynin treatment decreased BP and protein loss, and it improved renal morphology at least partly through the downregulation of desmin expression without changing FN and HIF-1α. Hyperbaric oxygen therapy improved hypertension but failed to significantly affect the level of proteinuria. Combined treatment (apocynin and HBOT) normalized blood pressure (BP) values, renal function, and improved kidney structure by modulating FN and HIF-1α, without affecting desmin protein expression. Further studies are needed to elucidate the mechanisms of slowing down the progression of CKD in this experimental model.

## 1. Introduction

Chronic kidney disease (CKD) is a progressive pathological condition, recognized as the presence of a decrease in the value of glomerular filtration below the value of 60 mL/min/1.73 m^2^, and/or the presence of at least one indicator of renal function impairment (albuminuria, for example), lasting for at least three months [1,2]. It is a serious condition, the prevalence and incidence of which have been growing [1,3] at an extremely high rate in the last few decades [4]. From a pathophysiological point of view, CKD is a complex condition, which results in the complete fibrosis of the kidneys, i.e., replacing almost completely functional parenchymal with fibrous tissue [5]. However, the mechanisms of CKD progression and fibrogenesis remain unclear. A large number of different animal models of CKD exist; however, none of them manage to fully provide a clear insight into the pathogenic mechanisms by which this condition develops, nor do they mimic the phenotypic characteristics of CKD in the human population [6]. The remnant kidney tissue model that is quite often used nowadays, especially for the investigation of fibrogenesis, is the 5/6 Nephrectomy (5/6 Nx) model [5,7].

Apocynin (APO) is an active substance isolated from the roots of the plant *Picrorhiza kurroa*, which has been used in Southeast Asia for several centuries as an ingredient of tonics with certain therapeutic roles [8]. It is a substance whose mechanism of action is still not entirely clear [9]. However, what is known is the fact that it is a substance that reduces the amount of reactive oxygen species (ROS) in the tissue while also having anti-inflammatory properties. Most studies mention the inhibition of the NADPH oxidase enzyme as the main mechanism of action [8,9,10]. Based on numerous studies of the mechanisms underlying the development of CKD and the mechanisms of action of apocynin [11,12,13,14,15], we hypothesized that apocynin, alone or in combination with other treatments, may have a protective effect on kidney structure and function in already developed CKD, induced by 5/6 Nx with the direct ligation of the poles (5/6 Nx-L).

Hyperbaric oxygen therapy (HBOT) is a therapeutic exposure of an organism to 100% oxygen in an environment with an increased atmospheric pressure value [16]. HBOT acts therapeutically by increasing the oxygenation of the blood and, consequently, the oxygenation of the tissues, thus preventing the development or treating already developed hypoxia [16]. Because there is an increasing interest in expanding the use of this treatment to a greater number of pathological conditions from others [17], as well as from our group [9,18] and knowing that hypoxia plays a role in the progression of fibrotic process in CKD [19], we hypothesized that HBOT can contribute to the prevention of the impairment of this pathological condition.

The aim of this study was to examine the isolated effects of apocynin and HBOT, as well as the effects of their combined application in the prevention of the progression of CKD and fibrogenesis, based on the 5/6 Nx-L model. Therefore, we estimated blood pressure, level of proteinuria, renal morphology, and specific target protein expressions in this experimental model, in response to the mentioned treatments.

## 2. Materials and Methods

### 2.1. Animals

Male Wistar rats were bred at the Institute for Medical Research, National Institute of the Republic of Serbia, University of Belgrade. The animals were housed under standard conditions (12 h light/dark cycle, 55 ± 10% humidity, 23 ± 2 °C temperature) with ad libitum access to water and food (standard chow for laboratory rats, Veterinarski zavod, Subotica, Serbia). The experimental protocol was approved by the Ethics Committee of the Institute for Medical Research, National Institute of the Republic of Serbia, University of Belgrade and Veterinary Directorate of Ministry of Agriculture and Environmental Protection of the Republic of Serbia (No. 323-07-07496/2022-05/1). This study was in accordance with the National Law on Animal Welfare (“Službeni Glasnik” No. 41/09) that is in harmony with the guidelines for the use of laboratory animals for experimental research purposes of the European Convention for the Protection of Vertebrate Animals Used for Experimental and Other Purposes (Official Daily N. L 358/1-358/6, 18 December 1986) and in strict accordance with the European Directive on the protection of animals used for scientific purposes (Directive 2010/63/EU of the European Parliament and of the Council, 22 September 2010).

### 2.2. Experimental Protocol

Animals (6–7 weeks old, with an average body weight of 135 g) were randomly divided in 5 groups (n = 8 per group). Control group—sham-operated rats; Nx-L group—rats with 5/6 Nx-L; APO group—rats with 5/6 Nx-L, treated with apocynin (20 mg/kg/day, single dose, i.p.); HBOT group—rats with 5/6 Nx-L, treated with hyperbaric oxygen (one session per day: 10 min gradual compression, 100% oxygen at 2ATA for 60 min, 10 min gradual decompression; 5 days per week for 20 sessions total, with no treatments on weekends); and APO+HBOT group—rats with 5/6 Nx-L, treated with both apocynin and hyperbaric oxygen. As previously described, 5/6 Nx-L was performed in two-step surgical procedure on anesthetized rats [3]. Briefly, the first step included the right flank incision; removal of the kidney after the ligation of the blood vessels and the ureter at the renal hilum (non-absorbable 1/0 Silk suture, JOST, AKOMED d.o.o; Belgrade, Serbia); and the closure of the abdominal cavity with suture (absorbable 3/0 PGLA quick, JOST, AKOMED d.o.o; Belgrade, Serbia). The second step was performed after 7 days; through a left flank incision the kidney was visualized, and approximately 1/3 from the upper and lower poles were ligated with non-absorbable suture (1/0 Silk, JOST, AKOMED d.o.o; Belgrade, Serbia). For ligation to be successful, the following 2 criteria had to be met: the circumference of the ligated pole was ½ of the circumference of the kidney’s remnant tissue, and the appearance of the complete discoloration of the kidney poles (due to ischemia). The ligated kidney was placed back in the abdomen and the incision was closed with absorbable suture. The control rats were sham-operated (both kidneys were only visualized through flank incisions which were closed with suture). All treatments of animals started 4 weeks after the second step. The duration of this period was determined on the basis of the pilot-study we conducted, as well as on the basis of data from studies performed by other authors [3,20]. This period was long enough for the development of CKD, which was particularly important to avoid any potential prevention of CKD with our treatments and lasted for the next 4 consecutive weeks. HBOT was conducted in a custom-made hyperbaric chamber (Holywell Neopren, Belgrade, Serbia), and calcium carbonate crystals were placed inside to reduce the CO_2_ generated during each session. The dose of apocynin and the HBOT protocol were selected from previous studies based on their safety and effectiveness in other models [21,22,23,24,25,26,27,28]. At the end of the treatment period, the rats were placed in individual metabolic cages for the 24 h urine collection. Urine samples were centrifuged (4 °C, 4000 rpm, 20 min) and stored at −20 °C until further analysis. Animals were weighed and anesthetized (sodium pentobarbital, 35 mg/kg, i.p.) for the measurement of the hemodynamic parameter. After the sacrifice of the rats, the kidneys were harvested and weighed; one half was formalin-fixed and the other half shock-frozen in liquid nitrogen and stored at −80 °C.

### 2.3. Hemodynamic Measurements

Mean arterial pressure (MAP) was measured by a catheter (PE–50, Clay-Adams, Parsippany, NY, USA) placed in the femoral artery, which was connected to a physiological data acquisition system (Cardiomax III-TCR, Thermodilution Cardiac Output, Columbus, OH, USA).

### 2.4. Biochemical Analysis

Protein concentrations in urine samples were measured by COBAS INTEGRA 400 plus (Hoffmann-La Roche, Roche Diagnostics GmbH, Sandhofer Str. 116, 68305 Mannheim, Germany). Protein urine excretion (Protexc) was used to assess proteinuria, expressed in mg/min/kg.

### 2.5. Western Blot Analysis

Samples of kidney tissues (6 animals per group) were homogenized in RIPA buffer, as previously described [29]. Equal amounts of protein were separated by SDS-PAGE and transferred to nitrocellulose membrane. Membranes were blocked with 5% non-fat dry milk (Sigma Aldrich, St. Louis, MO, USA) dissolved in TBS-Tween, and incubated with the following primary antibodies: desmin (1:500, ab15200), fibronectin (FN) (1:1000, ab2413), hypoxia-inducible factor-1α (HIF-1α) (1:2000, NB100-479), and glyceraldehyde-3-phosphate dehydrogenase (GAPDH) (1:2500, ab9485). Incubation with horseradish peroxidase-conjugated secondary antibodies followed, after which a chemiluminescence reagent was applied. Bands were visualized by ChemiDoc Imaging system (Bio-Rad Laboratories, Inc., Hercules, CA, USA) and analyzed in Image Lab v6.0.1. software. There were at least three independent immunoblot experiments carried out for each examined protein expression.

### 2.6. Histopathological Examination

Kidney tissues were fixed in 4% formaldehyde, dehydrated in alcohol, embedded in paraffin block, and cut into 5 μm thick sections. Slides were stained with Periodic Acid-Schiff (PAS) and Masson’s trichrome and observed under a light microscope BX53 with DP70 camera (Olympus, Hamburg, Germany) by two independent pathologists blinded to the experimental protocol. For the purpose of the semi-quantitative analysis, the whole surface of the middle cross-section was analyzed in the kidneys of all experimental groups, applying all the available magnifications in order to precisely evaluate even slight changes in kidney parenchyma, while microphotographs were taken under 100× (indicated with scale bar 200 µm), 200× (indicated with scale bar 100 µm), and 400× magnifications (indicated with scale bar 50 µm). Considering the different experimental protocol used in this study, we applied a similar but slightly modified scoring system compared to that previously described [29]. For the analysis of the degree of glomerular and tubulointerstitial alterations, we evaluated the capsular adhesion of the glomerular tuft, sclerotic changes in glomeruli, atrophy of tubular epithelium, interstitial fibrosis, and mononuclear infiltration as follows: not detected—grade 0; detected in less than 5% of affected structure—grade 1; detected in 5–10% of affected structure—grade 2; detected in 10–25% of affected structure—grade 3; and detected in more than 25% of affected structure—grade 4 (Table S1). A histopathological kidney injury score represented the sum of the observed changes.

### 2.7. Immunohistochemical Analysis

Immunohistochemical analysis was performed on 5 μm thick paraffin-embedded sections, which were subjected to deparaffinization, hydratation, and heat-induced antigen retrieval in citrate buffer (pH 6.0). Peroxidase and protein blocks were applied prior to incubation with a primary antibody for 1 h at room temperature. The following primary antibodies were used: desmin (1:100, ab15200), FN (1:100, ab2413), and HIF-1α (1:200, NB100-479). Secondary antibodies (UltraVision Quanto Detection System kit, Thermo Scientific, Waltham, MA, USA) were applied and the antigen–antibody reaction was visualized by 3,3′-diaminobenzidine (DAB). Negative controls were performed for each kidney sample by omitting the primary antibody (Figure S1). Desmin, FN, and HIF-1α had internal positive controls in each immunohistochemically stained sample, since we evaluated areas where desmin, FN, and HIF-1α are normally expressed in kidney tissue (Figure S2).

### 2.8. Statistical Analysis

Data are presented as the mean value ± standard deviation (SD). One-way analysis of variance (ANOVA) with the Fischer LSD test for multiple comparisons was used to determine the statistical significance of differences in the values of the parameters between groups (IBM SPSS Statistics software, v24.0, IBM, Armok, NY, USA). A *p*-value less than 0.05 was considered statistically significant.

## 3. Results

### 3.1. Blood Pressure, Urinary Protein Excretion, and Kidney Structure

**Figure 1:**
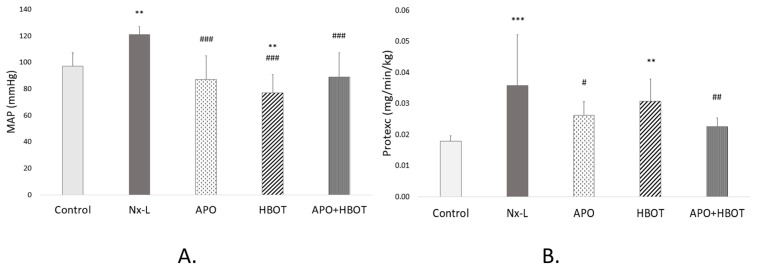
Effects of apocynin and hyperbaric oxygen therapy on blood pressure and level of proteinuria through different groups. Mean arterial pressure (MAP) (**A**); Urinary protein excretion (Protexc) (**B**). Values represent the mean ± standard deviation (n = 8); one-way analysis of variance (ANOVA), with the Fischer LSD test for multiple comparisons, was used to determine the statistical significance of differences in values of the parameters between the groups. **—*p* < 0.01; ***—*p* < 0.001; vs. control. #—*p* < 0.05; ##—*p* < 0.01; ###—*p* < 0.001; vs. Nx-L. Control—sham-operated animals; Nx-L—induced chronic kidney disease (CKD); APO—induced CKD, treated with apocynin; HBOT—induced CKD, treated with hyperbaric oxygen therapy; APO+HBOT—induced CKD, treated with apocynin and hyperbaric oxygen therapy.

Our results showed that the value of MAP was significantly increased in the Nx-L group compared to the control group (*p* < 0.01) (Figure 1A). Still, all treatments significantly decreased MAP compared to the Nx-L group (*p* < 0.001). Also, in the HBOT group, the MAP value was significantly lower compared to the control (*p* < 0.01). As for urinary protein excretion, it was higher in the Nx-L group than it was in the control, and this was significant (*p* < 0.001) (Figure 1B). Treatment with apocynin significantly reduced protein loss, while APO+HBOT treatment showed an even stronger decrease when compared to the Nx-L group (*p* < 0.05 and *p* < 0.01, respectively). HBOT treatment, however, slightly decreased the value of this parameter in comparison to Nx-L, but it was still significantly higher than in the control (*p* < 0.01).

**Figure 2:**
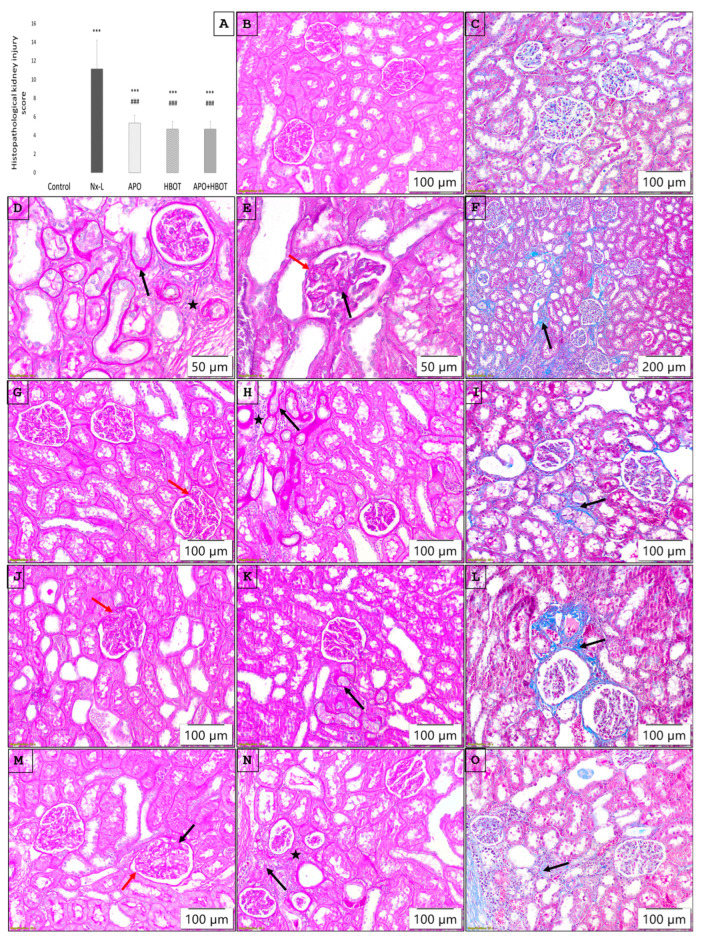
Pathohistology of kidneys in experimental groups: (**A**)—histopathological kidney injury score, presented as a sum of scores based on glomerular and tubulointerstitial alterations and expressed as a mean ± standard deviation; one-way analysis of variance (ANOVA), with the Fischer LSD test for multiple comparisons, was used to determine the statistical significance of differences in the values of the parameters between the groups; ***—*p* < 0.001 vs. control; ###—*p* < 0.001 vs. Nx-L; (**B**) Control group (PAS staining) with normal morphology of glomeruli and tubulointerstitium; (**C**) Control group (Masson’s trichrome staining) with normal morphology of glomeruli and tubulointerstitium; (**D**) Nx-L group (PAS staining) with a focus on atrophic tubuli (black arrow) surrounded by mononuclear inflammatory infiltrate (black star); (**E**) Nx-L group (PAS staining), with a focus on glomerulus with capsular adhesion (red arrow) together with slight segmental mesangial sclerosis (black arrow); (**F**) Nx-L group (Masson’s trichrome staining) with focus widespread interstitial fibrosis, visible as blue areas in interstitium (black arrow); (**G**) APO group (PAS staining) with a focus on glomerulus with capsular adhesion (red arrow); (**H**) APO group (PAS staining) with a focus on atrophic tubuli (black arrow) surrounded by mild mononuclear inflammatory infiltrate (black star); (**I**) APO group (Masson’s trichrome staining) with a focus on mild interstitial fibrosis, visible as blue areas in interstitium (black arrow); (**J**) HBOT group (PAS staining) with a focus on glomerulus with capsular adhesion (red arrow); (**K**) HBOT group (PAS staining) with a focus on atrophic tubuli (black arrow) without mononuclear inflammatory infiltrate in interstitium; (**L**) HBOT group (Masson’s trichrome staining) with a focus on mild interstitial fibrosis, visible as blue areas in interstitium (black arrow); (**M**) APO+HBOT group (PAS staining) with a focus on glomerulus with capsular adhesion (red arrow), together with slight segmental mesangial sclerosis (black arrow); (**N**) APO+HBOT group (PAS staining) with a focus on atrophic tubuli (black arrow), surrounded by mild mononuclear inflammatory infiltrate (black star); (**O**) APO+HBOT group (Masson’s trichrome staining) with a focus on interstitial fibrosis, visible as blue areas in interstitium (black arrow). Control—sham operated animals; Nx-L—induced chronic kidney disease (CKD); APO—induced CKD, treated with apocynin; HBOT—induced CKD, treated with hyperbaric oxygen therapy; APO+HBOT—induced CKD, treated with apocynin and hyperbaric oxygen therapy.

The histopathological examination of the renal tissue of control animals showed normal glomerular and tubulointerstitial morphologies (Figure 2B,C). In the Nx-L group, focal glomerular sclerosis with capsular adhesion of the glomerular tuft was present, along with tubulointerstitial lesions that included focally present fibrosis and mononuclear inflammatory infiltrate with tubular atrophy; the observed morphological changes were more pronounced in glomeruli (Figure 2D–F). In the APO (Figure 2G–I), HBOT (Figure 2J–L), and APO+HBOT (Figure 2M–O) groups, a lower extent of alterations was found. Although in all treated groups lesions were detected, as illustrated in Figure 2, the cumulative amount was significantly different, and it was represented by the histopathological score (Figure 2A) as a sum of these changes, showing a significantly higher sum in the Nx-L group compared to the control (*p* < 0.001). All examined treatments significantly decreased the intensity of lesions in the kidney compared to those observed in the Nx-L group (*p* < 0.001).

### 3.2. Desmin, FN, and HIF-1a Protein Expression in the Kidneys

**Figure 3:**
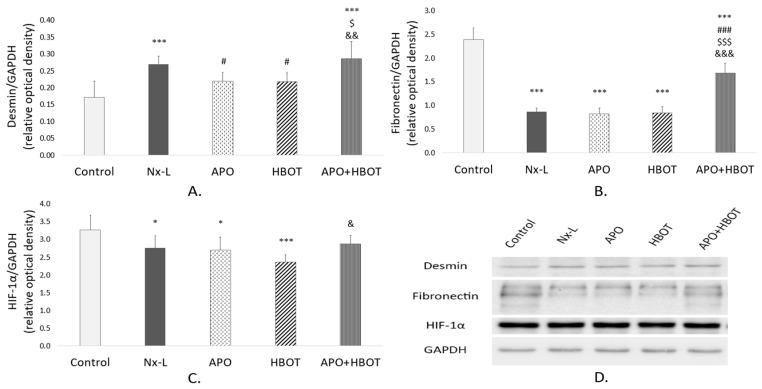
Desmin, fibronectin (FN), and hypoxia-inducible factor-1α (HIF-1α) protein expressions in the kidneys of animals between groups. The analysis of desmin/GAPDH (**A**), fibronectin/GAPDH (**B**), and HIF-1α/GAPDH (**C**) with the respective Western blots (**D**). There were at least three independent immunoblot experiments carried out. Values represent mean ± standard deviation; one-way analysis of variance (ANOVA), with the Fischer LSD test for multiple comparisons, was used to determine the statistical significance of differences in the values of the parameters between the groups. *—*p* < 0.05; ***—*p* < 0.001; vs. control. #—*p* < 0.05; ###—*p* < 0.001; vs. Nx-L. $—*p* < 0.05; $$$—*p* < 0.001; vs. APO. &—*p* < 0.05; &&—*p* < 0.01; &&&—*p* < 0.001; vs. HBOT. Control—sham-operated animals; Nx-L—induced chronic kidney disease (CKD); APO—induced CKD, treated with apocynin; HBOT—induced CKD, treated with hyperbaric oxygen therapy; APO+HBOT—induced CKD, treated with apocynin and hyperbaric oxygen therapy.

Our results showed that desmin protein expression in the kidney tissue of the Nx-L group was higher when compared to the control, and this result was significant (*p* < 0.001) (Figure 3A). Isolated treatments with apocynin and HBOT induced a significant decrease in desmin expression compared to that in the Nx-L group (*p* < 0.05). However, after combined treatment (APO+HBOT), desmin protein expression remained similar to that in the Nx-L group, and at a significantly higher level than that in both APO and HBOT groups (*p* < 0.05 and *p* < 0.01, respectively).

As for FN, interestingly, our results showed that the amount of FN in kidney tissue was significantly lower in the Nx-L group in comparison to the control (*p* < 0.001) (Figure 3B). Isolated treatments with apocynin and HBOT did not affect FN protein expression compared to Nx-L group, while combined treatment induced a significant increase in renal FN expression compared to the Nx-L, APO, and HBOT groups (*p* < 0.001), even though it was still significantly lower than in the control (*p* < 0.001).

HIF-1α protein expression was significantly lower in the Nx-L group of animals when compared to the control group (*p* < 0.05) (Figure 3C). Isolated treatments with apocynin and HBOT did not significantly change HIF-1α expression compared to the Nx-L group, while HBOT induced even a greater decrease in HIF-1α protein expression when compared to control values (*p* < 0.001). However, after combined treatment, HIF-1α protein expression increased to a level that is not significantly different from the control, and to a significantly higher level than that after isolated HBOT treatment was carried out (*p* < 0.05).

### 3.3. Immunohistochemical Detection of Desmin, FN, and HIF-1a in the Kidney

**Figure 4:**
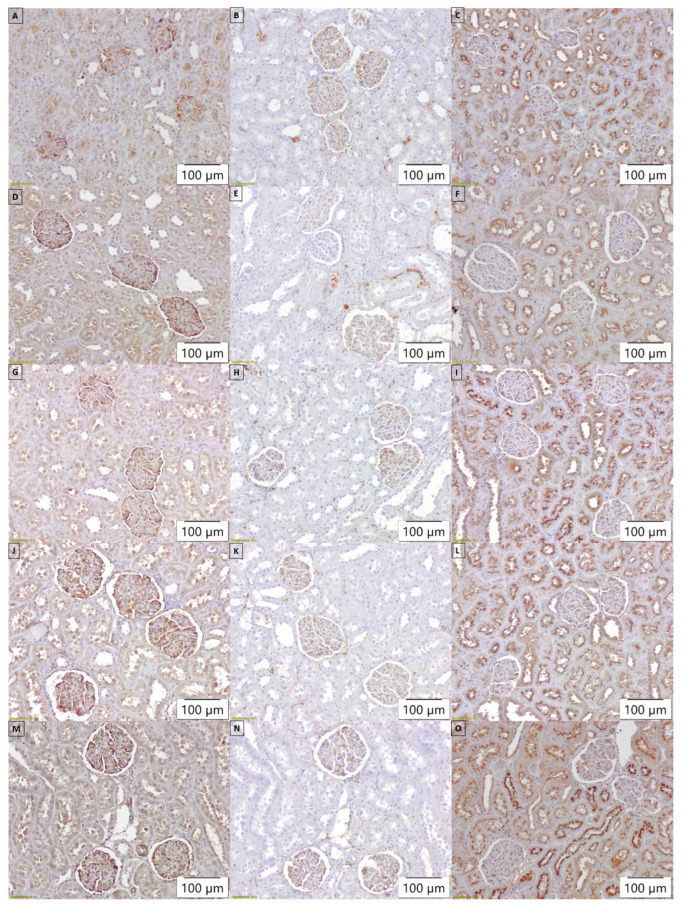
Immunohistochemical expression of desmin (left column—(**A**,**D**,**G**,**J**,**M**)), fibronectin (middle column—(**B**,**E**,**H**,**K**,**N**)), and hypoxia-inducible factor-1α (right column—(**C**,**F**,**I**,**L**,**O**)), in representative kidney samples, collected in different experimental groups as follows: Control (1st row—(**A**,**B**,**C**)), Nx-L (2nd row—(**D**,**E**,**F**)), APO (3rd row—(**G**,**H**,**I**)), HBOT (4th row—(**J**,**K**,**L**)), APO+HBOT (5th row—(**M**,**N**,**O**)). Scale bar = 100 µm. Control—sham operated Wistar rats; Nx-L—induced chronic kidney disease (CKD); APO—induced CKD, treated with apocynin; HBOT—induced CKD, treated with hyperbaric oxygen therapy; APO+HBOT—induced CKD, treated with apocynin and hyperbaric oxygen therapy.

Immunohistochemical staining of the kidneys of control rats showed normal desmin expression in glomerular cells and structures such as podocytes and mesangial cells, segmentally present in hyperperfused areas and blood vessels (Figure 4A). The stronger and more pronounced staining of desmin, especially in podocytes, was detected in the kidneys of rats in the Nx-L group (Figure 4D). In the APO (Figure 4G) and HBOT (Figure 4J) groups, a less prominent expression of desmin was observed in the studied renal structures. However, after combined treatment in the APO+HBOT group, we found a similar pattern to that of the Nx-L group (Figure 4M).

In the kidneys of control animals, FN was present in glomeruli (in rare podocytes, mesangial matrix, and glomerular basement membrane), and in interstitium, within the extracellular matrix (ECM) surrounding peritubular capillaries (Figure 4B). In the Nx-L group, we found a decreased intensity of FN expression across the same structures, especially in the glomerular basement membrane (Figure 4E). After isolated treatments with apocynin and HBOT, a similar pattern of FN staining was found as in the Nx-L group (Figure 4H,K). However, the combined treatment induced the improvement of FN expression in the examined renal compartment (Figure 4N).

HIF-1α staining in the kidneys of control rats was detected with moderate intensity, mostly in distal tubular cells (in cytoplasm) and with apical accentuation on the luminal membrane of the proximal tubular epithelial cells; also, HIF-1α expression was present in the glomeruli (Figure 4C). In the Nx-L group, HIF-1α staining decreased, especially in the glomeruli (Figure 4F). In the APO and HBOT groups (Figure 4I,L), similar patterns to those of the Nx-L group were found. However, after combined treatment (APO+HBOT group), we observed that HIF-1α staining pattern was closest to that previously observed in the control group (Figure 4O).

## 4. Discussion

In this study, isolated apocynin and HBOT treatments decreased MAP and the expression of desmin in the kidneys of nephrectomized rats, without affecting HIF-1α and FN expression. Further, both isolated treatments reduced renal structural changes, even though only apocynin decreased the level of proteinuria. Interestingly, after combined treatment, we found improved blood pressure (BP) values, kidney function and structure, in addition to the improvement of FN expression in the kidneys of Wistar rats with 5/6 Nx-L.

One of the most frequent findings in patients suffering from CKD is hypertension [30], which corresponds to the results obtained in our 5/6 Nx-L model, as well as in other studies [20,30,31,32,33]. Apocynin led to a highly significant decrease in MAP value compared to Nx-L. Similar results (decreased oxidative stress and hypertension in spontaneously hypertensive rats, attenuation of cardiac injury in cardiorenal syndrome, and improvement of cardiac remodeling in CKD leading to the normalization of hemodynamic parameters) have been obtained by several authors [34,35,36,37]. It is well known that ROS and reactive nitrogen species play an important role in the pathogenesis of hypertension in CKD [20]; thus, we presume that apocynin, an NADPH oxidase inhibitor, led to a decrease in BP values through the suppression of ROS generation [11,35] and the consequent reduction in peroxynitrite (^−^ONOO) formation [14]. HBOT in our study successfully reduced MAP compared to Nx-L. In addition, it caused a statistically significant decrease in MAP values even when compared to the control group. Contrary, other studies showed that HBOT leads to an increase in BP, and a decrease in heart rate values, most likely due to hyperoxia and consequent vasoconstriction, both in healthy individuals and in patients with various diseases (diabetes mellitus, hypertension) [38,39]. The same applies to animal research [40]. Still, Heyboer III et al. found that, with an increase in the number of HBOT treatments (long-term HBOT [28]), a protective effect of HOBT in terms of BP value exists [39]. Research suggesting the effect that HBOT exerts on BP values in CKD, in both clinical and experimental studies, are scarce. Nevertheless, we assume that the decrease in BP values in Nx-L animals exposed to HBOT may be related to the inhibition of sympathetic nervous system (SNS) activity (which plays an extremely important part in the pathogenesis of CKD [41,42,43]), but also in the reduction in the level of oxidative stress [20]. Studies that looked into the effect of HBOT on the cardiovascular system indicate an increase in the activity of the parasympathetic system as a consequence of HBOT [40]. In other words, it can also mean a decrease in SNS tone, all because of the changes in the activity of the baroreceptor reflex, caused by an initial increase in blood pressure [40]. It is possible that, after starting treatment with HBOT, there was an initial rise in blood pressure values in the animals in our experiment as well. However, that was a stimulus that initiated changes in the long-term activity of the baroreceptor reflex arc, with a consequent drop in pressure through a reduction in sympathetic activity [40]. In addition, short-term HBOT has been shown to increase the level of ROS due to the high partial pressure of oxygen in the blood and tissues [28]. However, long-term HBOT induces a very pronounced increase in the activity of antioxidant protection enzymes, which can actually lead to a net protective effect [28,44]. Also, as oxidative stress plays a big role in the progression of CKD, it is important to note that angiotensin II (ATII) promotes it by stimulating mesangial cells [20]. A decrease in sympathetic tone causes a decrease in ATII levels [42], so the stimulation of mesangial cells to produce ROS can also be decreased. At the same time, already created ROS are more efficiently removed by the activity of antioxidant protection enzymes, the activity of which is increased under the effect of long-term HBOT. Interestingly, the combined treatment failed to show a synergistic effect on MAP—the values are higher than in the HBOT group and approximately equal to the values in the APO group. The initial increase in ROS production after HBOT is what stimulates an increase in the activity of antioxidant protection enzymes, and thus, the powerful mechanisms by which the intensity of oxidative stress decreases [18]. It is possible that apocynin, by preventing this initial increase in ROS (by inhibiting NADPH oxidase [8,9,10]), removes the stimulus for production of antioxidant protection enzymes, so the results we obtained when combining these treatments corresponds to the results obtained in the group of animals treated only with apocynin. However, such a finding requires a larger number of studies examining the effects of these treatments, given that these are two different treatments that share similar mechanisms of action.

The results of our study showed that in rats with 5/6 Nx-L, the level of proteinuria was higher than in the control. Such results are in agreement with the results of a large number of studies, in which both this and many other animal models of CKD were used [5,20,33,43,45,46]. Proteinuria can be a consequence of pathological conditions at the level of the glomerular membrane and tubular damage [13]. Here, apocynin treatment decreased urinary protein loss, probably as a consequence of its antioxidant and anti-inflammatory properties, as shown previously [11,13,15,47,48,49,50]. On the other hand, proteinuria levels in animals exposed to isolated HBOT were not significantly lower than those in the Nx-L group, while they were still significantly higher than those observed in the control group. Such results can correlate with the results of the studies in which the relative independence of proteinuria on hypertension is recognized [43,51,52]. Contrarily, a retrospective study by Sedlacek et al. found that HBOT has a significant effect in reducing proteinuria levels in human patients with diabetic nephropathy [53]. Interestingly, combined therapy in our experimental model showed the most beneficial effect in reducing proteinuria, but future studies regarding the mechanism of APO+HBOT activity are needed.

CKD is characterized by the development of glomerulosclerosis and tubulointerstitial fibrosis [33,54], and these processes play a major role in the progression of CKD [5,54,55]. The same findings pronounced focal glomerulosclerosis and interstitial peritubular inflammation and fibrosis, as we have observed in rats of the Nx-L group in our study. In addition, an increased amount of desmin in kidney tissue is found in various CKD models [33,56,57], as we detected in 5/6 Nx-L. This increase in desmin expression during CKD development is a consequence of the presence of myofibroblasts, as they play a major role in the development and progression of fibrosis [55,58,59,60,61]. Also, injured podocytes [52,56,57] and mesangial cells [58,62], especially phenotypically altered ones, possess higher amounts of desmin [63]. These alterations, both at the glomerular [33] and at the level of tubules and peritubular interstitium, can be a consequence of chronic inflammation and oxidative stress, developed through ATII type 1 receptor-mediated signaling [5,52,54,64,65,66,67,68], promoting fibrosis. On the other hand, FN is a protein that is found in kidney tissue and represents one of the main components of the ECM of the mesangium, but it is also present in the peritubular interstitium [69]. Increased synthesis of the ECM protein FN is a characteristic of fibrosis [43,70,71,72,73,74,75]. However, it is interesting that the amount of FN is decreased in the Nx-L group. One of the explanations for such a result can be found in the results of studies in which it was shown that the composition of the ECM changes due to the internalization and degradation of FN by locally present cells, by means of an endocytosis process that is dependent on caveolin-1 (a protein that mediates processes such as endocytosis, autophagy, and oxidative stress [76]), and within which a very important place has α5β1 integrin as a receptor for FN. Inhibition of the endocytosis process, achieved by reducing the expression of caveolin-1, leads to a decrease in the degradation of FN [77,78]. Although we did not determine the level of caveolin-1 in the kidney, this could possibly explain the low level of FN in our study.

In rats of the Nx-L group, we detected a reduced renal HIF-1α protein expression, as found in several studies [79,80]. However, other studies indicate the opposite [81,82]. Such contradictory results are not surprising—the role of HIF-1α in the development of CKD has not yet been elucidated [79,80], despite the fact that kidney tissue hypoxia is recognized as a factor whose presence contributes to the development of CKD [19]. Several studies of CKD found that the degree of hypoxia is high at the level of preserved kidney tissue, while at the level of fibrosed tissue, hypoxia is almost absent, as the oxygen consumption by the cells of this tissue is low. In these regions, the level of HIF-1α expression was low [79,80]. Therefore, the occurrence of fibrotic changes in the Nx-L animals in our study, which was confirmed histopathologically, could be one of the reasons explaining the decrease in the expression of this protein in the kidney tissue of these animals.

Histopathologically, both isolated treatments as well as their combination improved kidney structure in our study. The effects of apocynin on the development of fibrotic changes and desmin expression in the kidney correspond to the results of previous studies. Liu et al. have shown that apocynin decreases the intensity of apoptosis (at the level of the epithelial cells of the proximal tubules), the ECM accumulation (at the level of the peritubular interstitium), and the transforming growth factor β expression (TGFβ), concomitant with the inhibition of NADPH oxidase activity and lower ROS production [49]. The same effect of apocynin on NADPH oxidase activity and ROS production has been noted by Cheng et al. on rats with unilateral ureteral obstruction (UUO), thus leading to a reduction in the number of myofibroblasts, and therefore, a reduction in the intensity of the fibrogenesis [65]. Although desmin levels were not determined in these two studies, the mechanisms that they suggested could have played a role in the increase in desmin in our study. Similar effects of apocynin on the fibrogenesis process are confirmed in previous studies [22,83,84]. Considering the beneficial effect of apocynin on fibrosis in the kidney, our result showed that the mechanism of action does not include FN and HIF-1α. However, other studies showed a decrease in FN expression in the kidney under the influence of apocynin in different models of CKD [85,86,87]. As for apocynin effects on HIF-1α, the tissue’s need for oxygen is decreased due to NADPH oxidase inhibition and the reduction in ROS generation [88].

Only a few studies investigated the effect of HBOT on fibrogenesis in renal tissue. Yuan et al. showed the protective effects of HBOT in models of pulmonary fibrosis, as evidenced by the decreased amount of collagen I, FN, α-smooth muscle actin, and the presence of myofibroblasts [89]. In animals with streptozotocin-induced diabetes, HBOT decreased collagen deposits and improved myocardial fibrosis by reducing the effects of TGFβ [90]. In our study, HBOT reduced glomerulosclerosis, interstitial inflammation, and fibrosis independently of FN and HIF-1α, indicating that the reduction in the intensity of fibrogenesis is related, at least partly, to the improvement in desmin protein expression. Under the influence of HBOT, the partial pressure of oxygen in the blood and tissues increases [28], which prevents the development of hypoxia. Song et al. showed a decrease in the amount of HIF-1α protein expression following HBOT in keloid scar tissue [91]. Taking into account the observations of Hung et al. and Faivre et al. [79,80], a reduction in the degree of renal fibrosis may lead to a consequent increase in metabolic needs and a greater tendency to hypoxia. Still, despite this, this treatment led to a decrease in the amount of HIF-1α compared to the control group. The amounts of this protein were also lower when compared to Nx-L, which, however, was not statistically significant. Combined apocynin and HBOT treatment successfully suppressed the nephrectomy-induced morphological changes to a similar extent as isolated treatments. Although the amount of desmin was similar to that obtained in the Nx-L group, combined treatment reduced kidney injury by the mechanisms that induced, at least partly, the improvement of FN and HIF-1α expressions.

## 5. Conclusions

In this study, we found hypertension, proteinuria, and renal morphological changes depicted in focal glomerulosclerosis, interstitial inflammation, and fibrosis in Wistar rats with 5/6 Nx-L. In addition, in this remnant kidney model, increased desmin was followed by decreased FN and HIF-1α expressions. Combined treatment (apocynin and HBOT) normalized BP values, renal function, and improved kidney structure by modulating FN and HIF-1α without affecting desmin protein expression. Apocynin treatment decreased BP and protein loss and improved renal morphology, at least partly through downregulation of desmin expression, without changing FN and HIF-1α. Hyperbaric oxygen therapy improved hypertension but failed to significantly affect the level of proteinuria. Regarding the notion that proteinuria and hypertension are major risk factors for the progression of CKD, the normalization of either of these conditions could slow down the progression of kidney disease. Further studies regarding the beneficial effects of the examined treatments are needed to elucidate the mechanisms of slowing down the progression of CKD in this experimental model.

### 5.1. Study Limits

Our study has several limitations. None of the currently known animal models of CKD fully match the phenotypic characteristics of CKD in humans. This is also the case with the 5/6 Nx-L model which was used in this study. Therefore, the translation of knowledge to human medicine is much more difficult. On the other hand, it is important to note that through the use of different models of 5/6 Nx, it was established that there are significant differences in the mechanisms and phenotypic characteristics of CKD in animals in which 5/6 Nx was performed in different ways. This makes the comparison of our results (obtained based on the 5/6 Nx-L model) with the results of other researchers, who used the 5/6 Nx model, much more difficult—either different 5/6 Nx methods were used, or it was not clarified which 5/6 model Nx was used in the given studies. To eliminate this type of problem in future research, it is necessary to increase the research pool using 5/6 Nx-L. Likewise, researchers need to be clear in stating the experimental model they use (whether conventional 5/6 Nx, 5/6 Nx with segmental artery ligation, or 5/6 Nx-L) due to the mentioned significant differences in a large number of parameters between animals in which 5/6 Nx was induced differently.

### 5.2. Perspectives

This study may contribute to the expansion of the spectrum of potential therapeutic treatments for CKD. In this sense, special mention is made to HBOT, given the increasing number of indications in which this type of treatment is used. Use of HBOT as a treatment of CKD can potentially provide a non-invasive and more affordable option for patients, which is crucial in the fight against this serious disease, especially in times when the number of patients suffering from this disease is growing rapidly.

## Data Availability

The data presented in this study are available within the article.

## Supplementary Materials

The following supporting information can be downloaded at: https://www.mdpi.com/article/10.3390/biomedicines12122788/s1, Table S1: Described scoring system used for histopathological evaluation of alterations observed in kidney samples, Figure S1: Negative controls for immunohistochemical staining, Figure S2: Positive controls for immunohistochemical staining ([92,93,94] are cited in Figure S2).
