# Supplementary material for: Apocynin and Hyperbaric Oxygen Therapy Improve Renal Function and Structure in an Animal Model of CKD
Source: Biomedicines. 2024 Dec 9;12(12):2788. doi: 10.3390/biomedicines12122788 (PMC11673868; doi:10.3390/biomedicines12122788)
Supplement: Supplementary file 1 [file biomedicines-12-02788-s001.zip › biomedicines-3326973-supplementary.pdf]

Supplementary Materials:

Table S1. Described scoring system used for histopathological evaluation of alterations observed in kidney samples.

Morphological changes	Grade description	Grade
**Glomerular alterations**		
Capsular adhesions	not detected	0
	less than 5% of glomeruli affected	1
	5-10% of glomeruli affected	2
	10-25% of glomeruli affected	3
	more than 25% of glomeruli affected	4
Segmental glomerulosclerosis	not detected	0
	segmental glomerulosclerosis in less than 5% of glomeruli	1
	segmental glomerulosclerosis in 5-10% of glomeruli	2
	segmental glomerulosclerosis in 10-25% of glomeruli	3
	segmental glomerulosclerosis in more than 25% of glomeruli	4
**Tubulointerstitial alterations**		
Tubular atrophy	not detected	0
	atrophy of less than 5% tubules	1
	atrophy of 5-10% tubules	2
	atrophy of 10-25% tubules	3
	atrophy of more than 25% tubules	4
Interstitial fibrosis	not detected	0
	fibrosis of less than 5% interstitium	1
	fibrosis of 5-10% interstitium	2
	fibrosis of 10-25% interstitium	3
	fibrosis of more than 25% interstitium	4
Interstitial mononuclear inflammation	not detected	0
	mononuclear inflammation of less than 5% interstitium	1
	mononuclear inflammation of 5-10% interstitium	2
	mononuclear inflammation of 10-25% interstitium	3
	mononuclear inflammation of more than 25% interstitium	4

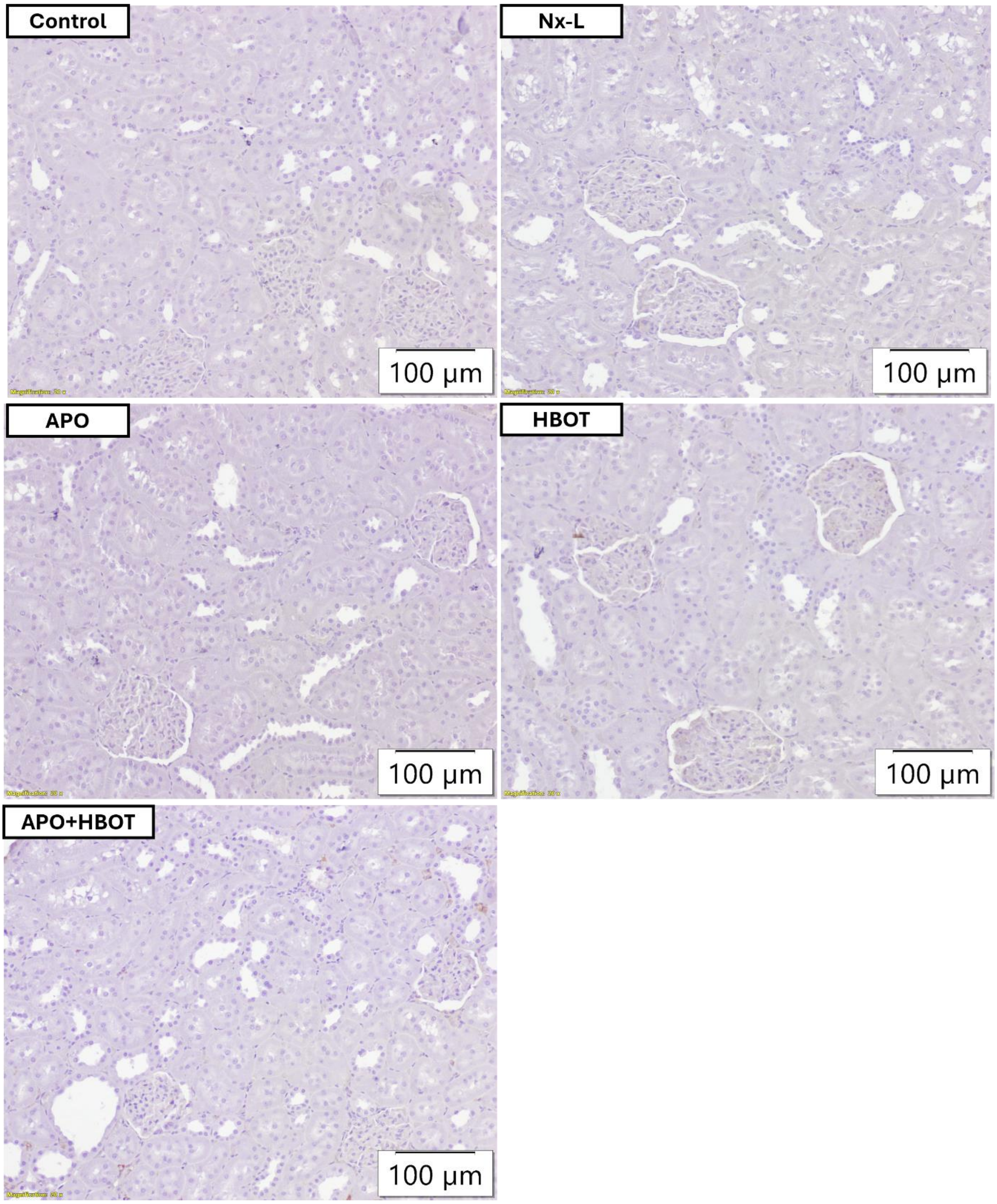

Figure S1. Negative controls for immunohistochemical staining. Magnification 200X. Control – sham operated Wistar rats; Nx-L – induced chronic kidney disease (CKD); APO – induced CKD, treated with apocynin; HBOT – induced CKD, treated with hyperbaric oxygen therapy; APO+HBOT – induced CKD, treated with apocynin and hyperbaric oxygen therapy.

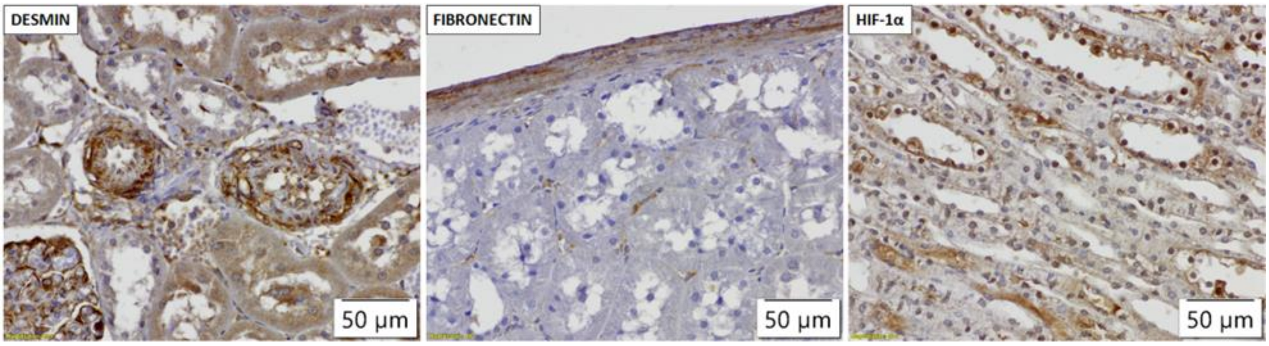

Figure S2. Positive controls for immunohistochemical staining. Magnification 400X. Desmin, fibronectin and HIF-1α had the internal positive controls in each immunohistochemically stained samples, since in kidney tissue desmin is normally expressed in smooth muscle cells in media of blood vessels [**92**], fibronectin is normally expressed in fibroblast-produced fibrous tissue, such as kidney fibrous capsule [**93**], and HIF-1α is normally expressed in kidney medulla in the deep portion of the inner medulla stripe in tubules [**94**].
